# Singing for Wellbeing: Formulating a Model for Community Group Singing Interventions

**DOI:** 10.1177/10497323221104718

**Published:** 2022-05-27

**Authors:** Natasha Hendry, Dr Siobhan Lynam, Caroline Lafarge

**Affiliations:** 1 Department of Psychology, 7364 University of West London, Brentford, Middlesex, UK; 2 School of Psychology, Social Work and Human Sciences, 7364 University of West London, Brentford, Middlesex, UK

**Keywords:** singing for wellbeing, singing-based interventions, group singing, community choirs, social prescribing, theoretical framework, person-centred social activity model, community health, social groups, social isolation, interpretative phenomenological analysis

## Abstract

Research into the benefits of community-based group singing, pertaining to positive wellbeing and Quality of Life is lacking. Additionally, no preferred theoretical framework exists for community singing-based interventions. For the present study, six members of a **UK community choir** were interviewed using a semi-structured interview approach. Interpretative phenomenological analysis (IPA) was employed. Analysis produced superordinate themes of: *Social Factors* with key elements such as social bonds and group identity; *Psychological Factors*, highlighting self-efficacy, self-identity and positive emotions and *Psychological Motivations for Joining the Group*, including autonomy, change of life circumstance and seeking a new challenge. The style/method of the group, teaching, music and group leader, were shown to have an influence on perceived benefits of the singing group. A key product of this study beyond the evidenced benefits of group singing is the development of an intervention model that optimises wellbeing outcomes in community singing groups underpinned by psychological theory, findings from the wider literature and the results of this study.

## Introduction

Some 2.14 million people sing regularly in choirs in the UK, with community choirs representing the largest sector at 36% (Voices Now, 2017). Stacy et al. (2002), in their review of the relationship between singing-based initiatives and health, concluded that singing had both physiological and psychological benefits. This has since been substantiated further by research in the two decades that have followed (Stegemoller, Hurt, et al., 2017; Weinstein et al., 2016). Physiological benefits proved easier to quantify in the 2002 review with a vast amount of research showing a positive relationship between singing and improved symptomatology while psychosocial benefits were harder to appraise.

### Physiological and Psychological Benefits of Singing

Research indicates that singing increases breathing patterns, reduces respiratory symptoms and encourages motivation to engage in a physical activity (Clift, 2010; Stacy et al., 2002). Singing also helps with various speech-motor abnormalities and swallowing control (Stegemoller, Hibbing, et al., 2017; Thompson et al., 2016; Wan et al., 2010). It is useful for pain management (Hopper et al., 2016) and enhances immune system activity (Beck et al., 2000; Beck, Gottfried et al., 2006; Kreutz et al., 2004; Kuhn, 2002). Music can also bring about other physiological changes such as lower blood pressure and pulse rates and increased oxygen saturation levels (Chan, 2007; Mok & Wong, 2003). While the documented physiological benefits of singing are numerous, many researchers pointed out that the main benefits relate to the positive impact on the quality of life (QoL) of participants and the enjoyment they experienced from singing (Camic et al., 2011; Stegemoller, Hibbing, et al., 2017).

Singing also has psychological benefits. Clark and Harding (2010) carried out a review of singing interventions for participants in therapeutic programmes and reported that active singing, particularly in a group, in comparison to just listening to music, is more beneficial to wellbeing and mood. Much of the research on singing for wellbeing has focussed on participants with specific health conditions, with fewer studies researching the potential health benefits of community-based singing, raising the question as to whether findings can be generalised to community groups. One exception is Judd and Pooley’s (2014) qualitative study on members of a community-based singing group who regularly participated in choral singing, to explore the psychological benefits of singing in a group. Results revealed benefits pertaining to the individual and the group such as increased positive emotions, facilitated mood change or expression of emotions as well as increased social networks and connectedness to others. Factors such as past experiences and type of choir and musical director seemed to influence the quality of experience and level of involvement participants would undertake. Associations between musical director and participant satisfaction were also made in an earlier study by Durrant (2005). Eells (2013) acknowledged that community-based research into singing is sparse but that singing was generally beneficial for social and personal health and wellbeing.

### Psychosocial benefits of Singing

Haslam (2018a) stated that social identity is also important for health, describing it as providing a sense of connection, meaning, support and agency. Hargreaves and North (1999) affirmed the importance of the social dimension of music in everyday life. They concluded that music has three main benefits to the individual’s psychosocial wellbeing pertaining to the management of self-identity, interpersonal relationships and mood. They argued that the social dimension should be at the core of music psychology and noted that until recently this aspect has been largely neglected. While Hargreaves and North’s study refers to music more generally, given that singing is a common form of musicking (defined as any musical activity including listening, performing etc.), these benefits may also be applied to singing. Self-identity as a psychosocial outcome of singing was addressed by Bannan (2000) who maintained that one way individuals can make sense of the world is through singing. He included positive elements of self-identity in his model for finding one’s singing voice, while Lamont (2002) explained that part of our identity is informed by music itself. A study by Dingle et al. (2013) on choral singing for marginalised populations suggested that becoming a choir member facilitates an additional social identity for members which could evoke positive emotions. Similarly, Lagace et al. (2016) found in their study of people living with mental illness involved in a community singing project, that improved self-perception and rediscovery of self-confidence were among the benefits reported.

### Singing for Community Health: Social Isolation, Depression and Stress

A growing problem in the UK is a lack of social connectedness. Nicholson (2012) reviewed research on social isolation between 1995 and 2010 and concluded that there is overwhelming evidence to suggest that social isolation poses a serious threat to health particularly for older adults, at the same time revealing a severe lack of viable interventions. Tomaka et al.’s (2006) study on older people reported that a sense of belonging correlates with good health outcomes. However, differences in findings for ethnic groups suggested a universal approach and outreach strategy for interventions is not adequate. For example, when comparisons between Caucasian and Hispanic participants were examined, family support was a more important factor for positive health outcomes for Hispanics, whereas belonging support outside the home was more important for Caucasians. However, Hispanic participants reported greater perceived social isolation despite greater family support. Camic et al. (2011) carried out a mixed methodological study on group singing and QoL of people with dementia and their carers. The quantitative study revealed no significant impact of the intervention on QoL; however numerous examples of a positive effect were found in the qualitative data set. This study indicates that results based solely on quantitative measures could be misleading when reporting on QoL and group singing, in addition to the fact that there does not appear to be one preferred standardised measure of QoL used in this type of research (Banerjee et al., 2009). Haslam (2018b) suggested that social isolation and loneliness is not just a problem for older people and that it affects all ages and demographics of society, those in good health as well as those in poor health. Family practitioners reportedly spend 20% of their time with patients addressing non-health issues with two thirds raising issues of social isolation (Caper & Plunkett, 2015).

Social connectedness and depression appear to be linked. Depressed individuals, in comparison to the wider population, belong to far fewer social groups and are shown to experience a 63% reduction in relapse across 2 years after joining a social group (Cruwys et al., 2013). Another prevalent psychosocial issue amongst the general population is stress. The largest and most comprehensive study of stress in the UK carried out on 4619 participants in 2018 reported that almost three quarters of the sample had at some point over the previous year, felt so stressed that they felt overwhelmed or unable to cope (Mental Health Foundation, 2018). Horenstein et al. (2018) found that stress is also a high predictor for several physical diseases. Their results led them to suggest that interventions to reduce susceptibility to anxiety are key in promoting better future health for individuals.

Art and health researchers have proposed that creative arts initiatives can offer much needed support for these social issues (Crozier, 1997; Davidson et al., 2014; Gabrielsson & Wik, 2003; Hays & Minichiello, 2005; Lee et al., 2010). Mok and Wong (2003) suggested that health and QoL can be improved by the relaxing and anxiety-reducing effects of music by ‘distracting from unpleasant experiences’ (p13). Decreased stress levels were also among the psychosocial benefits reported by choral singers in Judd and Pooley’s study (2014). Evidence suggests that group-based interventions are more successful than one-to-one formats (Dickens et al., 2011; Franck et al., 2016). The British Psychological society claims that ‘psychologists are unlocking the “social cure” afforded by groups, in a fine example of bridging the gap between research and practice, to influence public policy’ (Sutton, 2018, para. 2). A scheme announced by the British Health Secretary in 2018 (NHS England, 2018) proposed to enable UK doctors to issue social prescriptions, that is to offer patients creative arts and hobby-based solutions to their ailments (Solly, 2018).

### The Present Study

Singing is reported to be the most effective of all artforms in promoting wellbeing and good health (Morrison et al., 2007; Wall & Duffy, 2010). However, there is a gap in the literature concerning the experiences of community-based singing groups regarding the psychosocial wellbeing benefits of group singing and how best to harness them. In addition, there is a paucity of theoretical frameworks (Dingle, 2019) to account for and analyse such experiences. This study aimed to fill these gaps. It sought to learn how singing in a group affects community-based singing group members and to offer a comprehensive insight into their experiences and the psychosocial benefits they felt it afforded them. In doing so it aimed to enhance the understanding of the factors that facilitate these benefits and to develop a model for community singing interventions. The research question asked is: **What are the wellbeing effects of group singing among a community population?**

## Methodology

### Study Design

The study was concerned with people’s personal experiences, motivations and self-reported effect on their wellbeing as a result of being part of a singing group. The study adopted Dodge et al.’s (2012) dynamic and multi-dimensional definition of wellbeing. They proposed that a new definition of wellbeing should be centred around peoples’ perception of balance and equilibrium, appreciating that this concept can be affected by life circumstances and events. Given the subjectivity of concepts such as personal value and wellbeing, a qualitative approach using interpretative phenomenological analysis (IPA) (Smith, 1996) and semi-structured interviews for data collection was most appropriate to address the objectives. Interpretative phenomenological analysis has extensively been used in the field of health psychology and is concerned with understanding an individuals’ personal lived experience and the meaning they place on these experiences (Smith & Osborn, 2015). Interpretative phenomenological analysis also lends itself to subjective and under-researched topics (British Psychological Society, 2018). Interpretative phenomenological analysis has also been recognised as useful means of hearing directly from service users to inform health services within organisations such as the National Health Service (NHS) (Reid et al., 2005).

### Recruitment and Study Participants

Purposive sampling was used to recruit six participants from a local community pop choir led by the lead researcher. Due to the researcher’s close connection to the group, participants were encouraged to speak as freely as possible without censoring their responses and the second author was involved with data analysis to mitigate any researcher bias. The singing group was established in 2015 initially as an activity aimed at new mothers, though since its establishment the group has grown to include men, parents of older children and non-parents. At the time of the study, the choir membership was solely female. The group meets on a weekly basis for an hour and a half in the evening. Outside of rehearsals the singing group takes part in local events and live performances and has also produced video recordings of some of their arrangements. The choir leader is a professional singer and vocal coach with over 25 years experience of working in the music industry and is educated in music and psychology to post-graduate level.

Participants were recruited following an announcement at one of the weekly rehearsals, requesting volunteers to talk about their experiences of membership to the singing group. It was explained that only a handful of respondents were required so not everybody would be chosen to take part. The group was informed that chosen interviewees would be privately contacted. Neither wellbeing nor any other area of interest of group membership was specified to potential participants to avoid biasing their responses. The researcher chose six participants from the 10 who responded to the invitation. They were chosen to reflect the variation in group demographics and membership duration. An announcement was made in rehearsals the following week to explain the full quota of interviewees had been met and had been contacted. No disappointment was expressed by any volunteers who were not chosen.

Participants of the study were all female and aged between 38 and 70 years, with a mean of 48.5 years. Three participants were self-employed, two employed and one retired. Names of participants were changed to protect their identity in accordance with the British Psychological Society’s (BPS, 2018) code of ethics (See Table 1).

**Table 1.** Participant Information.

Participant#	Name (Pseudonym)	Age	Occupation	Time in Choir in years
1	Nancy	52	Architect/Artist	3.5
2	Sharon	47	Self-employed	2
3	Paula	45	Accountant	3.5
4	Verity	70	Retired	3
5	Kamila	39	Criminal case manager	2
6	Hayley	38	Freelance HR consultant	1

### Data Collection

Individual semi-structured interviews were used to collect data. The questions were designed to allow the participants to describe their experiences in a way that was unrestricted and personal to them. Questions were developed by addressing in a broad sense what membership of the singing group meant to them, without a focus on health or QoL to allow for a bottom-up approach. Careful structuring of the questions ensured that they were not leading in any way and allowed for participants to reflect positively or negatively on any subject. Full details of what the interview would involve were verbally conveyed to participants prior to its commencement. In addition, participants were presented with a participant information sheet which outlined the purpose of the study, what it would entail and important ethical details concerning their data, risks involved and confidentiality and anonymity. Permission to take part and to have the interview audio recorded was also given by signing a consent form. The study took place in London between 2018 and 2019. In accordance with the five steps of IPA, the first interview was transcribed using the recording, reflected upon, read and re-read and analysed before any further interviews took place. An initial interview was carried out with one participant as a pilot study. Pertinent revelations occurred during the pilot study that helped inform the rest of the data collection, for example, more prompts and a wider selection of questions were included. Each interview lasted between 49 minutes and 1 hour. The pilot interview was included in the final data set.

The interviews were largely led and shaped by the participants and the researcher was vigilant to allow the participants to take the interviews in the direction they wished to go whilst keeping to the relevant subject matter with suitable prompts. At the end of the interview participants were presented with a debrief sheet outlining important information such as contact details should they wish to view their transcript or the completed study, as well as a link to further information on singing, the Arts and wellbeing. An example of the type of questions asked include, ‘Why did you choose to join a singing group over any other group activity? What are the main factors that keep you attending the singing group? What does it mean to you to be a part of the singing group?’

### Data Analysis

As recommended with the IPA method (Smith & Osborn, 2015), analysis was conducted on a case-by-case basis. The first three steps of IPA analysis were followed for each interview after they were recorded before moving on to the next; that is to transcribe interviews, read transcript several times and identify themes/create table of themes. Themes were identified by the first author using IPA methods which involved firstly making initial notes in the margin of the document to draw out some of the ‘sense-making’ and then returning to the beginning of the transcript to document emerging themes from the initial notes all the while treating the entire transcript as one data source (Smith, 2015). The second author checked emerging themes. There was overall concurrence of researcher opinion. The emerging themes were then analysed using the traditional method of collating the statements and phrases that made up the emerging themes and grouping them together in clusters of similarity. These cluster groups were then transferred to a table of themes which detailed three superordinate themes with several subordinate themes for each interviewee. Finally, a master table of themes (see Table 2) based on all participants was created by making connections and associations between each participants’ table of themes. The hermeneutic circle (Smith & Osborn, 2015) was considered when analysing the data in that the parts, the whole and the dynamics of how these relate to each other was at the forefront of the process. This was apparent when viewing the detailed sections of an individual’s transcript and how those details illuminated their overall experience, as well as when reflecting on one participant’s experience next to another’s and how that informed the emerging themes and overall conclusions. The final two steps of IPA, writing up the results and a final statement, were undertaken after analysis of all six interviews was completed, as detailed above.

**Table 2.** Superordinate Themes and Related Themes.

Superordinate Themes	Themes	Example Participant Quote
**Social factors**	Group dynamics	*‘I feel on the outside of that group which isn’t the same thing as outside of the choir’. – Verity*
	Social bonds	*‘there is that closeness that develops with the people that you sing with’ – Kamila*
	Group identity	*‘I think I have found like a tribe of like-minded people and I feel like I belong* – *Paula*
**Psychological factors**	Learning a new skill	*‘having a regular skill that you are learning week by week. You are seeing personal development, you know’ – Kamila*
	Positive emotions	*‘I always come out feeling lighter and happier and positive’ – Sharon*
	Self-efficacy	*‘I guess I grew in confidence over the year’ – Hayley*
	Self-identity	*‘I’ve said before I’ve discovered or rediscovered things about myself’ – Nancy*
**Psychological motivations for joining group**	Autonomy	*‘I didn’t realise what I got out of it was really, finding my voice’ – Paula*
	Change of personal circumstance	*‘I was a full-time mum at the time, and I was keen just to have something that I could do that just wasn’t all about the girls’ – Hayley*
	Seeking a new challenge	‘I was looking for new challenges’ *– Sharon*

## Findings & Discussion

The aim of this study was to learn about individuals’ experiences of belonging to a community singing group in relation to wellbeing. Findings illustrate that members of this singing group experienced extensive positive psychosocial health outcomes, the extent of which were influenced by the group environment, the type of teaching methods, genre of music and leadership style. Motivations for joining the group appeared to be psychological needs experienced by this population. Three superordinate themes were identified as best conveying participants’ experiences: *Social Factors, Psychological Factors and Psychological motivations for joining the group*. Style and method *of group, teaching, music and leader* were found to influence all superordinate themes (See Table 2). The researchers used psychological theory to explain and analyse the experiences of participants.

When looking at the experience of being in a singing group using the findings of this study, Self-Determination Theory (SDT) (Deci & Ryan, 1985) is of interest. The evidence suggests that group singing meets the three basic psychological needs for humans as described by this model. That is, autonomy, a need to be master of one’s life, competence, a need to develop skills and mastery over tasks of importance to an individual and connection/relatedness, a need for a sense of belonging and relatedness to others (Deci & Ryan, 2000). SDT also refers to types of motivation. These include intrinsic motivations, where self-determination and competence are important such as engaging in an activity for no apparent ‘outward’ reward, but one’s own internal satisfaction: and extrinsic motivation, for example, group affiliation, approval and rewards including recognition by others (Deci & Ryan, 2000). This theory and others relevant to the findings are discussed as we examine each superordinate theme.

### Social Factors

This theme captured relational factors which emerged from the data and although some intragroup conflict was evident, the related constituents mainly illustrate social benefits to the individual as a result of being part of the singing group. Although data were collected before the COVID-19 pandemic, findings in this superordinate theme have relevance for communities today who have been starved of social connection as we navigate the fallout of the pandemic. This study provides evidence of the ability of group singing to meet a variety of social needs including connectedness, and personal and group identity, supporting existing literature on singing group interventions where beneficial social factors such as connectedness, improved social networks and social engagement are well documented (Clark & Harding, 2010; Eells, 2013; Judd & Pooley, 2014).

#### Group dynamics

Group dynamics were complex with acknowledgement of an in-group (core members, long serving, highly involved in singing and social activities) and out-group (newer members or those attending infrequently and non-participatory in social activities). Many participants referred to the in-group as the core group. Participants identified this group as those that were there for social reasons as well as the singing: *‘I think there will always be some kind of core group because there’s that external socialisation as well*’ (Verity). Although some intragroup conflicts did impact people’s enjoyment of the group at times, most felt that interacting with many different types of people was a worthwhile and growth-enhancing experience and the social aspect was often what pulled them through group difficulties. In this way, group dynamics were shown to nurture a positive social outcome from group singing, bringing people together, from different walks of life, who otherwise may not have interacted. Paula reflected this when she explained:

You know singing together with them, has already like given you something in common with them and suddenly you’re like talking to people who are like outside of your regular type of people you would normally mix with.

Findings from this study suggest that group dynamics do need to be managed as they have the potential to undermine the psychosocial benefits of group singing. Findings also show that a choir is not only helpful for increasing social networks but for helping people manage interpersonal skills as was also asserted by Hargreaves and North (1999).

#### Social bonds

Social bonds were illustrated by a growing sense of community facilitated through the shared experiences the singing group provided. Nancy described feeling a *‘Sort of togetherness’* when commenting on the group. Crozier (1997) emphasised the social role of music and it came across as a prominent theme in the interviews. All but one participant referred to friendships as a strong and additional benefit of membership of the singing group. Participants highly valued the social benefits the group added to their quality of life alongside the singing itself. Reflections made in the interviews demonstrated that for most, on initial membership to the group, the singing itself was most important but as time passed and friendships formed, the social aspect became as valued as the singing:

*I’d say its 50-50…it’s of equal* – *not importance – but of equal kind of like, for satisfaction if you like. I very much enjoy the singing, but I enjoy the social part as well.* – Sharon

Group cohesiveness was strengthened by the combination of singing together and socialising together. Social interaction and greater social connectivity are described as psychosocial benefits of group singing in the literature (Hays & Minichiello, 2005; Lee et al., 2010) and while this is supported in the findings of this study, participants’ experiences also pointed to something more unique about the nature of singing together as a uniting force. Nancy described this cohesiveness in terms of synchronised breathing of the same air:

you know we’re breathing the same air, and once a week when we are in choir and we’re all sort or um singing and we’ve got to blend our voices and with breathing in at a certain time and breathing out at the same time and we are sort of, er, I don’t know there’s a sense of community.

Camlin et al., (2020) recently referred to the many ‘paramusical benefits’ (more than just music) of group-singing which involved a complex entanglement of musical, neurobiological, psychosocial and spiritual experiences, for which particpants often struggle to find language for. Singing is also a more effective group activity than others with respect to social connectivity, Paula explained: *‘I have made friends faster doing performances in choir than I did like going for years to the same class at the gym’.*

#### Group identity

Group identity was an important theme, manifested through a feeling of connectedness with others, a sense of belonging and ownership of the group as well as increased self-esteem via the group accomplishments. The group identity served to boost individual self-confidence as people were given the courage to step out and try things within the safety of the group and referenced a kind of safety in number*s: ‘having the support of the group around you…knowing these people have got your back’ – Paula*. Participants expressed a strong sense of ownership and belonging to the singing group that suggested an emotional attachment:

I felt like it belonged to me, I had been there for since four or six weeks from the beginning, it’s like it belongs to me, I belong to it. – Paula

Social and group identities were important in the singing group and appeared to facilitate a sense of belonging and self-esteem. The findings suggest that singing in a group involves personal investment that reaps benefits for the group and the individual in an enriching way. Social Identity Theory (SIT) (Tajfel & Turner, 1979) is manifested throughout the data as members were seen to gain self-esteem from the positive social identity derived from membership of the group. It is observed that participants’ social identity in the group was stronger than their self-identity in some instances, as noted in comments made by Hayley:

If I had the guts to go and stand up and sing a solo, it would sound mediocre but if get up and sing with lots of people, I am actually part of something amazing.

Self-affirmation theory (Steele, 1988) posits that we seek to publicly affirm a positive aspect of oneself in order to conceal other aspects of self that appear less positive. Research from Howell (2017) suggests that self-affirmation in this way has a positive effect on wellbeing and increases self-efficacy and self-control, both important components for improving mental and physical health (Foster et al., 2016; Matthews et al., 2016). One participant described using the identity she finds within the group as a positive temporary replacement of her own identity ‘I think maybe, maybe I just felt like a little celeb for just an hour a week’ – Hayley.

Haslam (2018a) also emphasised the importance of social identity for health to provide meaning and a sense of connection to individuals. Although it could be argued that positive social identity could be enforced by any group activity, this concept is supported amongst the group singing literature (Dingle et al., 2013). The data do suggest that group singing affords some specific beneficial factors to individuals pertaining to self-esteem and identity, interpersonal skills, a deeper level of connectivity and positive emotional attachment to the group. The findings represented in this theme related to social factors suggest that singing as an activity is more beneficial in a group setting than on an individual basis.

### Psychological Factors

Consistent with other research on group singing (Clift, 2010; Eells, 2013; Judd & Pooley, 2014; Reagon et al., 2016), this study revealed considerable psychological benefits associated with communal singing. Psychological related themes appear to contribute to improved psychological wellbeing and QoL for participants, this in turn appears to have a positive impact on people’s self-esteem. High self-esteem has been shown to be important for health (Marmot, 2003) in a variety of ways including associations with greater life expectancy (Fitzpatrick, 2001) and with depression when self-esteem is low (Cheng & Furnham, 2003).

### Learning a new skill

Learning a new skill in singing allowed individuals to engage in a challenge and gain a sense of accomplishment and fulfilment as described by Kamila, *‘when you do pick up a skill you do feel boosted, you do feel that your head CV* [mental skillset] *is growing’*. Findings corroborate evidence in the literature which suggests that learning a new skill is linked to increased self-esteem (Leman et al., 2012). The sense of fulfilment and achievement derived from their improved singing ability was considered a positive part of self-development by participants. Gaining a level of competence in a task described as meaningful, satisfies one of the three basic human needs according to Self-determination theory, SDT (Deci & Ryan, 1985). This theme was interrelated with the motivations for joining the singing group theme, as many people cited the desire to take on a new challenge as a main motive for membership.

#### Positive emotions

Positive Emotions were among the most reported positive outcomes from being in the singing group. Many participants repeatedly used terms such as a general sense of ‘feeling good’, ‘positive’ and ‘on a high’ when making reference to what they gained from singing group membership, in fact, all but one used the term ‘on a high’. Paula summed up the general feeling of the group: *‘it has given me happiness that I didn’t expect that I would have’.*

This finding supports other research in the literature, which links positive emotions with being in a singing group (Hargreaves & North, 1999; Judd & Pooley, 2014). Other references to the positive emotions referenced the singing groups’ stress-relieving properties: *‘I guess it’s relaxing*’ – Sharon, and the escapism it provided from daily stress: *‘it sounds cheesy to say that you can sing your worries away, but you can’* – Sharon. Singing seemed to be used as a coping strategy for stress by many of the participants:

The whole balance shifted at home – suddenly Wednesday, singing in a choir which was my one uplifting thing, became that much more important, cos at home the general mood level had liked dipped. – Paula

Kamila also found attendance at the singing group helpful at a particularly stressful time in her life caring for a seriously ill child:

Even when Jack was like in intensive care, when he was in hospital, there would still be some weeks that I would come …because it just helps you then cope with whatever else is going on in your life, whatever that might be.

The coping strategies provided by the singing group for participants worked in accordance with the transactional stress theory (Lazarus & Folkman, 1987) and were emotion-focused and problem-focused, in that they reduced the anxiety and fears of external stressors as well as offered important social support. The general ability to enhance or change mood afforded by singing in the group was prominent in the data set. This changed mood state seemed far-reaching and long-lasting: *‘just the anticipation of going into choir boosts me up you know. Wednesdays set me up for the rest of the week’* – *Paula*. Evidence from this study is supported by the substantial literature on the ability of music and singing to increase mood ratings (Clark & Harding, 2010; Judd & Pooley, 2014; Kreutz et al., 2004). These findings are important because improved wellbeing is associated with positive emotions (Fredrickson, 2001).

#### Self-efficacy

Self-efficacy was another prominent psychological benefit seen in the data set. Not only did participants feel enabled in the task of singing but many also reported having a greater belief in their own abilities in other areas of life. The confidence boost reported by many members did not seem to just benefit people at the time of singing but something of the positivity it generated appeared to stay with people and permeate their lives in other areas. Participants testified to feeling more confident in their homes as parents, in their workplace roles and felt a general feeling of capability to take on other challenges and tasks. Paula’s account testifies to this:

I started to feel more confident as a parent as well. It was er, it was quite eye-opening…It has given me the confidence to stand up and speak in front of people…if I can stand on a stage and sing in front of like a hundred people, then I can talk in a board meeting to six to eight directors sitting in front of me who don’t know much about figures, I can do that now.

Improvements to self-confidence are documented in the literature on community singing (Lagace et al., 2016) and extensive research points to the importance of high self-efficacy for improved health outcomes (Foster et al., 2016; Matthews et al., 2016). Although generally self-efficacy is seen to be impacted in a positive way throughout the data set, participants reported that it can be negatively affected at times. Comparison and competition within intragroup relationships was said to cause damage to self-efficacy as well as group cohesiveness. Again, the importance of managing group dynamics to increase the potential for psychosocial benefits is highlighted (Judd & Pooley, 2014).

#### Self-identity

The final psychological factor to emerge was self-identity. Self-identity was seen to be positively impacted by membership of the singing group, as individuals reported it to provide congruence to changed personal and social status, as well as retrieving a part of self that was deemed lost: *‘my whole identity was about my career, so when I was suddenly at home with no career, I lost some identity’* – Paula. Sharon commented: *‘.it almost feels like the old me*’. Being part of the singing group was an element of how people self-identify: ‘*it’s become a part of my life*’ – Nancy. Self-identity was found to have positive associations with music by Hargreaves and North (1999) and this study corroborates those findings as well as the resulting increase in self-esteem.

The importance of self-identity is emerging as a strong factor for wellbeing in the study of positive psychology. Research in this area is showing a positive impact on mental and physical health outcomes. Miquelon and Vallerand (2008) posit that self-realisation, as they term it, promotes physical health by producing positive coping strategies that consequently reduce stress in people’s lives, such as engaging in activities conducive to personal growth, in this case seen to be the singing group. They also posit that self-realisation encourages more vigilant and less avoidant styles of coping (Miquelon & Vallerand, 2006). Self-identity as a theme also interacts with the self-efficacy constituent as reports of boosted confidence are linked with a positive self-identity brought about via membership of the singing group:

So *basically, it was like finding a new confidence, ….I really lost confidence in myself and lost the identity of I am this career woman, this accountant, so actually coming to choir actually really built up my confidence.* – Nancy

### Motivations for Joining the Group

This study also enquired about participants’ motives for joining a singing group and in doing so revealed various psychological needs. Interestingly, only two of the six participants described wanting to sing specifically as a motive for joining the group. On examination of the related themes, the participants’ needs seemed consistent with the basic human psychological needs described by Deci and Ryan’s Self-Determination Theory (1985). In line with this theory, participants in this research mostly displayed intrinsic motivations for joining the singing group: that is engaging in the activity for reasons of satisfaction in itself as well as for the personal developmental and growth-enhancing properties it affords. O’ Connell and Cuthbertson (2009) also claim people join groups to meet self-actualisation goals.

#### Autonomy

This theme revealed that many participants’ motives for joining the group were born from a desire to gain back some control over how they spent their time. Many participants reported a lack of time for themselves and a desire to do something outside of their work and family constraints, therefore, the singing group was a way to get back to the self. Numerous studies have revealed that women spend less time on leisure than men and more time on housework and childcare compared to men (Bittman & Wajcman, 2000; Shaw, 1994; Wallace & Young, 2010). Kamila’s account summarised a sentiment expressed by most participants: *‘I wanted to do something that didn’t involve my job or my family, that was the reason. I wanted to do something for myself’*. Pursuing goals out of autonomous motives is positively associated with happiness and self-realisation (Miquelon & Vallerand, 2006). Shaw (1994) investigated women’s leisure and constraints and supported the concept that positive outcomes from activity engagement are in part dependant on the perceived degree of choice or self-determination. Paula, like other participants, referred to making a self-determined decision to make time for herself:

It forces me to make time for myself, to go to choir practice an hour and a half a week, it’s my time. Before that I never prioritised time for myself. I am always helping other people.

SDT’s ideas about autonomy as a basic human psychological need are clearly supported here, even if that need was not conscious for participants when they joined the singing group.

#### Change of circumstances

Participants testified to various changes in life circumstances ranging from personal trauma such as life-changing illness and miscarriage, to approaching new milestones in life such as becoming a parent or experiencing a career-break. All life events described impacted upon self-identity, highlighting an association with that theme: *‘my whole identity was about my career, so when I was suddenly at home with no career, I lost some identity’* – Paula. These life events had an effect on participants’ psychological health: *‘This actually plunged me into like a bit of a depression’* – Paula. It is also possible to see how these changes put some at risk of social isolation, evoking a desire for new social connections: *‘I had my life literally turned upside down through illness and with my illness I was in isolation, I was not allowed to see people’* – Sharon. Previous research supports that singing groups are useful for helping people through changes in life and recovery from illness (Judd & Pooley, 2014) and other adverse life events (Von Lob et al., 2010). Sharon testified to this: *‘I love it [choir] and having the experiences, it’s the positive things that have come out of the illness*’. Various changes in personal circumstances leaving people disconnected from previous social circles, roles and identities appeared to motivate them to search for a new sense of belonging and identity, in this way, the relatedness need of SDT is also highlighted within this theme.

#### Seeking a new challenge

Seeking a new challenge was another prominent motivation for joining the group, sometimes in relation to consciously wanting to resist social isolation and ‘get back out there’ and at other times was seen to be for self-developmental purposes. Kamila explained: *‘I am quite into building my character and doing something different, doing things out of my comfort zone, um seeing what comes of something’*, Nancy added: *‘I think I saw it as a bit of a challenge’*. This constituent highlighted participants’ intrinsic desire to engage in an activity that was stimulating, personally challenging and extra from their current activities. SDT’s competence need can be referenced here, concerned with an individual’s desire to build their achievements, knowledge and skills. Intrinsic motivations were again evident as people spoke about wanting to stretch themselves, reach new potential and face fears:

If it hadn’t been the choir I think I would have taken up something like ballroom dancing or I definitely wanted to do something that was slightly scary. – Nancy

Rogerian theory (Rogers, 1961) also came to mind when analysing the data that contributed to this theme. Rogers’ five characteristics of ‘the fully functioning person’ include, being open to experience, existential living, trusting own feelings, creativity and living a fulfilled life which he believed incorporated continuing to look for new challenges and experiences (McLeod, 2013). The needs highlighted in this superordinate theme, revealed via the participants’ motivations for joining the group, appeared to be met by the evidenced psychosocial outcomes from membership of the singing group. This study demonstrates possible needs in the general population and highlights the mechanisms which link singing and wellbeing which to date has been sparse in existing research (Clift, 2010).

### Mediating Factors: Style and Method

Style and method refer to the mechanisms of the group in terms of atmosphere, the music genre, teaching methods and the group leader. The study findings show that these elements acted as mediating variables to the psychosocial outcomes of group singing membership. The atmosphere generated by the style of the **singing group** itself was important in terms of members feeling welcomed, safe and accepted: Hayley observed: *‘I think that people are already comfortable around each other…I think it is very nurturing’*. Clift’s (2010) research showed the importance of the singing group providing a safe, non-judgemental environment. Accessibility and inclusivity were thought to be high in part due to the no-audition policy, *‘there was no pressure, no audition, no pressure to commit to anything and you don’t have to be the best voice out there.’* – Sharon. The style of **music** for some was unimportant or became insignificant next to the other benefits reaped and the fact that it seemed possible to make even disliked songs and styles sound pleasing in the group context: *‘I don’t feel it* [the song choice] *but that is the weird thing, suddenly when you are singing with everyone and there is such a good buzz and a good energy you get into it’ –* Kamila. However, others commented: *‘the feeling of singing, being part of a song that you really like that is definitely better’* – Hayley. Few negative effects have been noted on the use of music as an intervention, but music can evoke unwanted bad memories according to some research (Hays & Minichiello, 2005). Kamila made comments supporting this finding: *‘the music can take you to places … songs always remind us of things – good or bad’*.

The **teaching** methods were praised for being inclusive, in that it was learning-by-ear, so not a requirement to be able to read music. This generated a feeling of self-efficacy towards the task of singing: ‘*the other thing that I was aware of was I didn’t want to join one of the very structured choirs because I knew that my sight reading was short’* – Verity. The personability of the group **leader** and their lead in making the group a fun, welcoming, safe, but organised experience was also considered important and further facilitated feelings of competence in the members: ‘*I think it’s important again because it’s a confidence thing, I don’t feel judged* [by the choir leader]*’* – Sharon. Commenting on the choir leader Sharon explained:

It’s nice to have an element, a sort of professionalism in it for me. It’s nice to have fun and think you can have fun and have a laugh as well, but also it is professional.

Judd and Pooley (2014) reported that ‘The MD [musical director] affects the overall tone of the choir via their personality, musical ability, teaching style and their vision for the group’ (p. 281). Durrant (2005) referred to the importance of the nurturing qualities of the musical director and reported that choirs were motivated by both the musical and interpersonal skills of the group leader. Judd and Pooley (2014) also found in their research on community choirs that other factors such as style of music and type of choir affected individuals’ choice of singing group and their sense of fulfilment.

Deci and Ryan proposed a sub theory to SDT called Cognitive Evaluation Theory (CET), which states that intrinsic motivation can be undermining or facilitating dependant on influencing social and environmental factors (Deci & Ryan, 2000; 2008). They stated that communication and feedback that produces feelings of competence when performing an activity will encourage intrinsic motivation for that activity. CET also maintains that high levels of intrinsic motivation are fulfilled by feelings of self-efficacy and personal development in an individuals’ environment. Similarly, Carl Rogers’ (Rogers, 1961) person-centred approach ascertained that certain core conditions must be provided so individuals can move towards personal growth and self-actualisation (McLeod, 2013). He described the core conditions as sufficient amounts of acceptance, warmth and congruence (McLeod, 2008). Based on these assumptions, the accounts of the participants support both theories. The style/method of group, music, teaching and leader were considered to be important factors for members to find the singing group a fulfilling experience and to help them remain motivated to be part of the group. In this way, the style and method factor provide key information about the conditions needed to best facilitate and meet the needs of the psychological and social factors detailed in the three superordinate themes. Both participants’ expectations and outcomes are influenced by this mediating factor making it an important finding for this and future research that seeks to inform interventions using singing-based activities.

## A Proposed Model for Intervention

It is proposed that singing groups could provide a viable intervention for promoting wellbeing in community-based populations and meeting psychological needs such as autonomy, connectedness, competence, positive self-identity and self-esteem. In addition, singing groups can also provide much needed support for public health issues such as stress, depression and social isolation. Using music and singing as a therapeutic intervention has been shown in the literature to be an inexpensive and safe method of promoting wellbeing and good health (Eells, 2013; Morrison et al., 2007). Cruwys (2018) emphasised, ‘An important next step for this research [on the association between participation in social groups and improved health outcomes] is to translate these various lines of evidence into useful, concrete interventions that increase social group connectedness’ p43. Clift (2010) emphasised that a weakness in the literature is the absence of a theoretical model which demonstrates how singing is associated with wellbeing. Using psychological theory evidenced in this study and the existing literature, the researchers have proposed a model for singing-based interventions.

### A need for an integrated model

Self-Determination Theory argues that it is not sufficient to meet just some of the three basic psychological needs, all must be fulfilled to facilitate wellbeing (Deci & Ryan, 2008). At the same time, research by Ryff et al. (2004) produced findings to show that self-realisation was associated with decreased perceptions of daily stress; however, happiness had no effect on perceptions of stress. The indication is that positive emotions alone are not enough to produce psychological resilience to stress, but that personal growth and self-development as received from facing new challenges and raised self-efficacy are necessary (Deci & Ryan, 2000; Lagace et al., 2016). This study and wider research demonstrate a need for an integrated theoretical model to meet multiple needs and harness as many of the psychosocial benefits that group singing can provide as possible. The researchers have integrated the psychological theories discussed in the literature, with the findings of this study, in a model that can be used as a framework to better understand how group singing can positively affect individual and community wellbeing. The model ascertains that when key elements of self-determination theory and cognitive evaluation theory, social identity theory (Tajfel & Turner, 1979) and the person-centred approach (Rogers, 1961) are adhered to, the psychosocial benefits of group singing are at their most powerful and effective. The combined theoretical concepts evidenced in the research come together to form the Person-Centred Social Activity model (PCSA), which can be used to inform recommendations for practice when working with group singing interventions.

### The Person-Centred Social Activity Model (PCSA)

**Figure 1.**
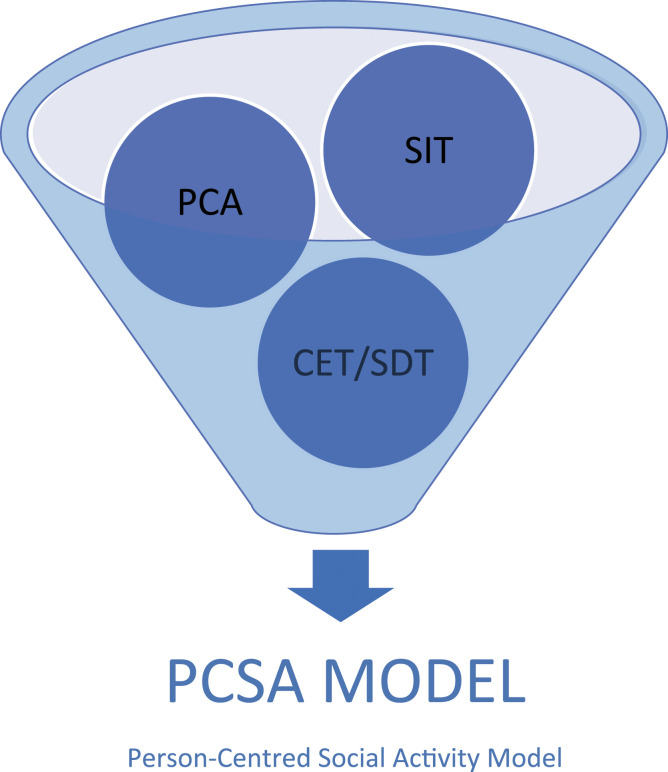
The theoretical components of the PCSA model (PCA: Person-Centred Approach, SIT: Social Identity Theory, CET: Cognitive Evaluation Theory, SDT: Self-Determination Theory).

A core assumption of the proposed model is that the provision of certain conditions within the environment, teaching style and leadership should reflect the components of self-determination theory and facilitate a sense of autonomy, belonging and competence for singing group members. If people feel empowered, enabled and have a sense of belonging, the wellbeing benefits of group singing may be seen. The model supports the cognitive evaluation theory’s proposal that high levels of intrinsic motivation are fulfilled by feelings of self-efficacy and personal development and that this is necessary for the activity to facilitate wellbeing (Deci & Ryan, 2000). The model also ascertains that, as proposed by social identity theory, being part of a singing group can provide a positive social identity which can be self-affirming, causing a positive effect on wellbeing and increasing self-efficacy and self-control. Finally, the model upholds Carl Rogers’ (Rogers, 1961) assertion that core conditions such as acceptance, warmth and congruence are important within the singing group environment and leadership to move individuals towards personal growth and self-actualisation. Figure 1 illustrates the theoretical components of the PCSA Model.

Some practical suggestions based on the PCSA model of how the relevant psychological theories can be used in a group singing context are detailed in Table 3. The study findings provide evidence of the psychosocial outcomes and support these recommendations.

**Table 3.** Recommendations for Singing Group Interventions Directed by the Supporting Psychological Theory and Literature on Singing Groups.

Recommendation	Based on psychological theory	Potential psychosocial outcome	Study/evidence for benefit to singing groups
Learning-by-ear rather than reading sheet music to increase accessibility for all and promote a can-do feeling for singers relatively quickly and easily	SDT/CET Deci & Ryan (1985)Deci & Ryan (2000, 2008)	Competence at a skill important, Increased self-efficacy, raised self-esteem	Clift (2010) Judd & Pooley (2014)
Ensuring members contribute to group decisions and the future direction of the group through regular feedback	SDT Deci & Ryan (1985)	Sense of autonomy, self-determination & belonging, positive group/social identity	Miquelon & Vallerand (2006) Shaw (1994)
Doing away with tie-in payment schemes and working on a pay as you go basis to give members control and intrinsic motivation to be there	SDT Deci & Ryan (1985)	Autonomy	Miquelon & Vallerand (2006) Shaw (1994)
Ensuring choir leaders are inclusive, approachable, encouraging and dedicated to making the experience fun as well as informative	CET, PCA Deci & Ryan (2000, 2008) Rogers, (1961)	Sense of belonging, acceptance, competence, facilitated personal growth	Camic et al. (2011) Clift (2010) Judd & Pooley (2014) Weinstein et al. (2016)
Making room for social activities for the group in and outside of the singing setting	SDT, SIT Deci & Ryan (1985) Tajfel & Turner (1979)	Connectedness/improved social networks, positive social identity, belonging, positive emotions	Camic et al. (2011) Weinstein et al. (2016)
Having a leader who can manage group dynamics, so group benefits are not undermined	PCA, SIT Rogers, (1961) Tajfel & Turner, (1979)	Positive social identity, acceptance, warmth, facilitated personal growth	Judd & Pooley (2014)
Having a welcoming atmosphere and no-audition policy	PCA Rogers, (1961)	Belonging, acceptance, warmth, increased self-efficacy, positive emotions	Camic et al. (2011) Clift (2010) Weinstein et al. (2016)
Be aware of the influence singing groups have on members’ self-perception & self-identity	SIT Tajfel & Turner, (1979)	Positive social identity, self-identity, positive emotions	Hargreaves & North, 1999 Lagace, Briand, Desrosiers & Lariviere (2016) Lamont (2002)

The PCSA model for group singing-based interventions makes the following recommendations (see Table 4).

**Table 4.** Recommendations for Practice Based on the PCSA Model.

Recommendations for Practice
• A group singing intervention should take a person-centred approach. Core conditions should be provided for security and growth/self-actualisation and to ensure the activity is facilitating intrinsic motivations not undermining them as explained by CET (Deci & Ryan, 2000, 2008; Durrant, 2005; Judd & Pooley, 2014; present study)
• A group singing intervention should satisfy all three of the basic psychological needs as proposed by SDT; autonomy, competence and relatedness/belonging and not neglect any one of them (Deci & Ryan, 2008; present study)
• A group singing intervention should recognise the importance of social identity and self-realisation for positive health and wellbeing outcomes as highlighted by the present study, Deci and Ryan (2000, 2008) and Miquelon and Vallerand (2006, 2008).
• A group singing intervention should be accessible and inclusive for all regardless of ability, confidence, or social status (Clift, 2010; Judd & Pooley, 2014; present study).
• A primary focus of a group singing intervention should be raising self-esteem for participants through increased competence and self-efficacy (Lagace et al., 2016; present study).
• Group dynamics should be carefully managed in a group singing intervention to facilitate and not undermine psychosocial benefits (Judd & Pooley, 2014; Reagon et al., 2016; present study).

).

This study had several limitations. The main researcher and interviewer was the leader of the choir featured in the study. This could have presented some response bias from participants with whom she had a personal relationship, as well as reporting bias due to the researcher’s vested interest. To attempt to reduce these biases, a second member of the research team reviewed the data and themes to provide a balanced analysis and detect any interview bias. A further weakness to this study is that it only includes women, so the perspective of men is neglected. Platt (2009) found trends for gender and race in social participation research. Bangladeshi and Pakistani men and women were less likely to seek organised activities outside the home than White or Indian counterparts. The same was true of Black African women. Risk of social isolation was generally higher for women across the board with Black Caribbean and Black African women experiencing the highest risk. Further study on specific ethnic minority groups, gender groups and other marginalised groups such as disabled and LGBTQ+ communities, should be considered for future research.

## Conclusion

Group singing interventions could provide a cost-effective preventative tool for both mental and physical ill health thus reducing financial, manpower and other pressures on health and social care systems. Consideration in social policy of the encouragement and facilitation of group membership, in particular to singing and music-based activities, should be given to promote wellbeing among the general public. This may be particularly significant as communities continue to navigate the COVID-19 pandemic and the subsequent mental and physical challenges it has bestowed. This study identified a gap in the research to bring together psychological theory and the literature on group singing to inform a model for community singing-based interventions. The study findings are transferable to other community group settings because similar to what has been found in previous studies (Dickens et al., 2011; Franck et al., 2016), the key components that evoke a sense of wellbeing are demonstrated to be centred around connectivity, a positive social identity and the sense of personal development brought about via learning a new skill in a person-centred environment. A clear insight into a typical community singing group is presented here from a rich and reliable data source. The data are reliable because the participants are submerged in the experience of being part of a community singing group. Participants shine a light on the many positive factors that keep them there, both expected and unexpected as well as due acknowledgement of the challenges that group-life can also bring.

The findings from this study support much of the existing research on group singing and wellbeing and existing psychological theory. Beyond the widely recognised positive emotions that music and singing engagement specifically evoke, this study demonstrates that other less obvious factors for wellbeing are involved, namely: the association between singing group membership and self-actualisation, personal and social identity and social connectedness, which are gaining more attention as important components for good mental and physical wellbeing (Haslam, 2018a; Miquelon & Vallerand, 2006, 2008). The study results also point to potential negative effects of group singing pertaining to challenges of group dynamics such as intragroup competition that can damage self-efficacy, self-esteem and group cohesiveness. These findings reveal important areas for future research to examine the connections between group singing and personal development as well as other possible negative effects.
